# Effects of Dietary Plant Protein Replacement with Insect and Poultry By-Product Meals on the Liver Health and Serum Metabolites of Sea Bream (*Sparus aurata*) and Sea Bass (*Dicentrarchus labrax*)

**DOI:** 10.3390/ani14020241

**Published:** 2024-01-12

**Authors:** Valeria Donadelli, Patrizia Di Marco, Alberta Mandich, Maria Grazia Finoia, Gloriana Cardinaletti, Tommaso Petochi, Alessandro Longobardi, Emilio Tibaldi, Giovanna Marino

**Affiliations:** 1 Italian National Institute for Environmental Protection and Research (ISPRA), 00144 Rome, Italy; valeria.donadelli@isprambiente.it (V.D.); mariagrazia.finoia@isprambiente.it (M.G.F.); tommaso.petochi@isprambiente.it (T.P.); alessandro.longobardi@isprambiente.it (A.L.); giovanna.marino@isprambiente.it (G.M.); 2 Interuniversity Consortium INBB—Biostructures and Biosystems National Institute, 00136 Rome, Italy; alberta.mandich@gmail.com; 3 Department of Agricultural, Food, Environmental and Animal Sciences (Di4A), University of Udine, 33100 Udine, Italy; gloriana.cardinaletti@uniud.it (G.C.); emilio.tibaldi@uniud.it (E.T.)

**Keywords:** liver histology, liver index, sustainable feed, poultry by-product meal, *Hermetia illucens* meal, plant protein, lipid metabolism, fish welfare, blood biochemistry

## Abstract

**Simple Summary:**

This study focuses on the use of high-quality and sustainable feed ingredients, derived from circular bioeconomy processes, as major components of new diets for Gilthead sea bream and European sea bass, the most important farmed fish in the Mediterranean basin. In this study, we investigated the effects of replacing dietary plant proteins with insect and poultry by-product meals on the liver health of sea bream and sea bass through histological and biochemical analyses. Four diets were tested, including a control fish meal-free diet, based on plant-derived ingredients, and three other ones, in which 40% of the plant protein was replaced with *Hermetia illucens* and poultry by-product meals. The results revealed distinct responses to the test diets between sea bream and sea bass, in terms of biometric parameters, lipid metabolism, and liver condition. Sea bream utilized dietary nutrients efficiently, adapting to the test diets in terms of weight gain, physiological well-being, and liver health. Conversely, the test diets led to significant hepatic lipid accumulation in sea bass, resulting in an increased risk to liver health.

**Abstract:**

The liver health of Gilthead sea bream and European sea bass, fed with fish meal-free diets, including various proportions of plant proteins, as well as insect and poultry by-product meals, was investigated through biochemical and histological analyses using a new liver index (LI) formula. Four isoproteic (45% Dry Matter, DM) and isolipidic (20% DM) diets were compared, including a plant-based control diet (CV) and three other test diets, in which 40% of a plant protein-rich ingredient mixture was replaced with meals from *Hermetia illucens* (H40) or poultry by-product (P40) alone, or in combination (H10P30). The trials lasted 12 and 18 weeks for sea bream and sea bass, respectively. The results obtained thus far highlighted species-specific differences in the physiological response to dietary changes. In sea bream, the biochemical and histological responses suggest favorable physiological and liver health statuses, with higher serum cholesterol (CHO) and triglyceride (TAG) levels, as well as moderate hepatocyte lipid accumulation, with the H10P30 diet compared to the CV (*p* < 0.05). In sea bass, all diets resulted in elevated serum TAG levels and lipid accumulation in the liver, particularly in fish fed the P40 one (*p* < 0.05), which resulted in the highest LI, coupled with a higher frequency of severe lipid accumulation, hypertrophy, cord loss, peripheral nuclei displacement, and pyknosis. In conclusion, sea bream adapted well to the test diets, whereas sea bass exhibited altered hepatic lipid metabolism leading to incipient liver steatosis, likely due to the high lipid contents of the diets, including the insect and poultry meals. The LI formula developed in this study proved to be a reliable tool for assessing the effects of dietary changes on the liver health of sea bream and sea bass, consistent with biochemical and histological findings.

## 1. Introduction

The world’s population is projected to reach around 8.5 billion people in 2030, and aquaculture is expected to provide approximately 53% of the world’s seafood supply, significantly contributing to global food security and sustainable development [1,2,3]. The global aquaculture production of fed species is projected to reach 58.9 million tons by 2025, with an estimated demand for 69.5 million tons of aquafeed [4]. To support this growth, the industry will need to explore innovative solutions for sourcing sustainable ingredients through which to increase aquafeed production [5]. Over the past two decades, the transition toward the use of plant-based ingredients to reduce dependence on fish meal and fish oil has been challenging [6,7]. In fact, high levels of substitution in feeds for carnivorous fish can lead to adverse physiological effects on digestibility, as well as nutrient utilization, growth, metabolism, gut integrity, immune response, disease resistance, and overall health and welfare, which are still under study [8,9]. Similarly, the shift toward alternative ingredients generated through the circular bioeconomy will require significant efforts to achieve a sustainable balance between food production and resource conservation, as well as to enhance resilience to climate change within aquafeed production and aquaculture farming systems [10,11,12,13].

Poultry by-product and insect meals have received significant attention as alternative protein sources for both fish and plant protein-rich meals. This is due to their characteristics associated with circularity, environmental sustainability, and minimal conflict for direct human consumption. Poultry by-product meals are cheap, easily available, and contain high nutritional value for carnivorous fish species, as they are rich in crude protein, with a nearly complete amino acid composition, and are sources of minerals and vitamins [14,15]. Among edible insect species, processed meals derived from the black soldier fly (*Hermetia illucens* Diptera order) are also rich in protein, with a high biological value and an essential amino acid profile comparable to those of fish meal [16,17,18,19]. They also contain lipids and bioactive compounds that provide beneficial effects on fish health by stimulating the immune system and modulating the gut microbiota [20]. Deficiencies in omega-3 fatty acids and some vitamins can be improved through the selection of high-quality feeding substrates [21]. Currently, insect meal production is still limited and expensive, but the intensification of industrialized insect production systems could significantly increase production volumes and reduce production costs to meet the demands of the aquafeed industry [22].

To date, many studies have examined the replacement of fish meal with poultry by-products and insect meals in various fish species [23,24,25,26]. For Gilthead sea bream, *Sparus aurata*, and European sea bass, *Dicentrarchus labrax*, the results obtained thus far have provided broad support for the utilization of both alternative protein sources as valid substitutes for fish meal to develop sustainable aquafeeds [27,28,29,30,31,32,33,34,35,36,37,38,39,40,41]. 

On the other hand, only a few research studies have evaluated the partial replacement of plant protein-rich feeds with insect and poultry by-product meals, singly or combined, in low-fish meal diets for sea bream [42,43] and sea bass [44]. These studies mostly investigated the dietary effects on appetite, growth performance, gut health, and fish quality, while fish response in terms of liver health status has received limited attention.

Indeed, in fish, as in other vertebrates, the liver plays a key role in energy metabolism and storage, including in the synthesis of bile, proteins, and hormones, as well as in immune defense and detoxification [45,46]. Due to its role in metabolizing products from the digestive tract, the liver is also considered a valuable marker of nutritional pathologies [47]. Therefore, histopathology, which provides an analytical view of diseases and their impacts on tissues and cells, represents a valuable tool in studies on fish nutrition, particularly in feeding trials aimed at evaluating the suitability of alternative ingredients for innovative aquafeed [48,49]. Histological qualitative and semi-quantitative methods for evaluating hepatic tissue alterations have been widely applied in farmed fish to assess the effects of different diets on liver health. General morphology, lipid deposition, and glycogen distribution in the cytoplasm have been described and considered as valid parameters for comparing liver status with respect to test diets in both sea bream and sea bass [28,50,51,52]. In other studies evaluating the tolerance of these species to fish meal replacement with novel dietary ingredients, a semi-quantitative scoring system was applied, and several parameters, including nucleus shape and nuclear displacement, hypertrophy, cytoplasmic lipid accumulation in hepatocytes, and peripancreatic fat infiltration, were evaluated using graded system scales [29,40,53,54,55,56,57]. Some studies applied the semi-quantitative Bernet’s protocol [58], an organ-by-organ evaluation system originally designed to monitor the health status of fish exposed to environmental pollution [59,60,61,62,63]. This protocol was subsequently adapted to evaluate diet-related alterations [64], the general health status of farmed fish [65], and the quality of their larval and juvenile stages [66]. The method used by Bernet is the subject of a recent review proposing standardization for histological evaluation to generate reliable, high-quality, and comparable data in fish histology [67]. 

Additionally, histological quantitative methods involving the analysis of morphometric parameters, such as hepatocyte area, hepatocyte nuclear area, and their relative ratios, as well as lipid droplet area, were frequently used as indicators of hepatocyte metabolic activity in several farmed fish species, including sea bream and sea bass [68,69,70,71,72,73,74]. 

The present study is part of a larger experiment, in which graded levels (10, 20, or 40% protein basis) of poultry by-product and *H. illucens* meals, either alone or in combination, were included in a fish meal-free diet to replace plant protein-rich derivatives and to diminish the adverse physiological effects associated with the use of these latter ingredients. In this study, we specifically focused on the effects of diets with the highest level of substitution on the liver health status of sea bream and sea bass. Ensuring a healthy liver condition is highly desirable in developing novel feed formulations for these commercially important Mediterranean fish species [75]. For this purpose, the histological semi-quantitative scoring system proposed by Bernet et al. [58] was modified by developing a new formula for calculating the liver index (LI) and its related scoring scheme. Complementary blood biochemistry analysis was performed to evaluate the nutritional–metabolic status of the fish. Additionally, body condition indices and gross liver anatomy were analyzed to provide a comprehensive assessment of liver health status and evaluate its potential consequences on fish physiology in response to dietary treatments.

## 2. Materials and Methods

### 2.1. Test Diets

Four test diets were formulated to be grossly isoproteic (45%), isolipidic (20%), and isoenergetic (21 MJ kg^−1^). A control diet, rich in plant-derived ingredients (denoted as CV), was designed to obtain a 90:10 weight ratio between the vegetable and marine protein and a 67:33 weight ratio between the vegetable and fish lipids. The weight ratios of ingredients were calculated from the crude protein and lipid contributions to the whole diet of marine- and plant-based feed ingredients. 

From the basal formulation above, three other test diets were prepared by replacing 40% of crude protein from the vegetable protein-rich ingredient mixture of the CV with an equal amount of partially defatted *H. illucens* prepupae meal (H40 diet; ProteinX™; Protix Company; Dongen, The Netherlands) or poultry by-product meal (P40 diet; Agricola Tre Valli s.r.l., Villaganzerla, Vicenza, Italy), or a combination of the two ingredients (H10P30 diet), while maintaining the same 67:33 vegetable-to-fish lipid ratio as in the CV. Marine derivatives, based on a mixture of squid meal and fish protein concentrate, were used at 5.5% concentration in all diets as feed-attractants/stimulants. Wherever necessary, pure essential amino acids were supplemented in the diets to meet the nutrient requirements of sea bream and sea bass (National Research Council (NRC), 2011). All diets were manufactured as extruded pellets (3 and 5 mm) by SPAROS Lda (Olao, Portugal) and stored in sealed plastic buckets in a cool room until used. Proximate composition analysis of the test diets was performed according to the AOAC methods [76]. All diets were analyzed for dry matter (105 °C for 24 h), ash via combustion in a muffle furnace at 500 °C for 5 h (Nabertherm L9/11/B170, Bremen, Germany), crude protein (N × 6.25) using the Kjeldahl system (K-350 Buchi, Fisher Scientific Italia; Segrate (MI), Italy), and crude fat according to Bligh and Dyer, as modified in [77]. Gross energy was determined in an adiabatic bomb calorimeter (Model Werke C2000, IKA, Staufen, Germany). The ingredients and proximate compositions of the test diets are shown in Table 1.

### 2.2. Fish Husbandry and Sampling

Two experiments on sea bream and sea bass were carried out at the aquaculture facilities of the Department of Agricultural, Food, Environmental and Animal Sciences of the University of Udine. 

For this study, a total of 216 juvenile sea bream (48.8 ± 8.8 g) and sea bass (44.0 ± 5.1 g) were randomly divided into 12 fiberglass tanks of 250 L each (18 individuals per tank) included in a marine recirculating aquaculture system. 

Water temperature and salinity were kept at 23.0 ± 1.0 °C and 29 ppt for both sea bream and sea bass, and fish were kept under a 12L:12D photoperiod. Fish were acclimated for two weeks to culture conditions and fed a commercial diet before being randomly assigned in triplicate to the four dietary treatments.

Fish were fed to satiety, twice a day for six days a week, using belt feeders. The trial lasted 12 weeks for sea bream and 18 weeks for sea bass, up to triple their respective initial body weights. All fish were sampled at the end of the experiment after 24 h fasting. 

Five fish per tank of both fish species (i.e., 15 fish per dietary treatment, n = 60 fish) were rapidly anesthetized with Tricaine methanesulfonate MS222 at a dose of 150 mg/L (PHARMAQ, Fordingbridge Hampshire, UK) and reached a deep stage of anesthesia within 3 min. Blood samples were drawn from the caudal vein and were centrifuged at 3000 rpm for 10 min at 4 °C after clotting. Serum aliquots were deep-frozen at −80 °C until analysis, according to Di Marco et al. [78].

Fish were then sacrificed with an overdose of MS222 at 300 mg/L and individual body weight (BW) and total length (TL) were recorded. Fish autopsy was performed by observing gross anatomy and the occurrence of macroscopic alterations. The livers of all sampled fish were observed both in situ and after dissection, before being weighed to the nearest 0.01 g. A low, medium, or high degree of lipid accumulation was assigned to each liver by two operators, based on gross diffusion of lipid depots, as well as changes in color and consistency. Liver samples were further collected from 6 specimens from each dietary treatment, fixed in Bouin’s solution for 24 h, washed, and preserved in 70% ethanol for histological analysis. 

Based on biometric data collection, fish condition indices were calculated as follows:

Fulton’s condition factor (K) = 100 × body weight/length^3^

Hepatosomatic index (HSI) = 100 × (liver weight/body weight)

### 2.3. Liver Histological Analyses

Small liver subsamples were dehydrated, clarified, and paraffin (Bio-Optica, Milan, Italy)-embedded, following standardized protocols for the examination of hepatic tissue morphology. For each liver, slides were prepared by mounting three sections (5 μm thick), at 100 µm intervals. Two slides were stained with Hematoxylin–eosin (H-E, Bio-Optica, Milan, Italy) for hepatocyte morphology, and one slide was stained with Periodic Acid Schiff (PAS; Bio-Optica, Milano, Italy) to highlight glycogen deposition in hepatocytes. Before the histological examination, coded slides were quickly checked under a light microscope at 100× magnification to exclude those presenting possible technical tissue artefacts. General histomorphology was first evaluated for the description of the hepatic tissue in all the specimens according to test diets. For histomorphometric analysis and histopathological evaluation, 9 fields (0.320596 mm^2^ each) were randomly selected in three liver sections under a DMLB microscope (Leica, Wetzler, Germany), and 9 microphotographs (216 for sea bream and 216 for sea bass, in total) were acquired with a Leica ICC50-HD camera (Leica, Wetzler, Germany) at 200× magnification. All histological observations were performed by experienced personnel in two independent, blinded evaluations. 

#### 2.3.1. Histomorphometric Analysis

Hepatocyte and nucleus area were measured (90 cells per fish) on microphotographs using the Image J 1.41 software [48], and the nucleus/hepatocyte (N/H) ratio was calculated. The degree of lipid accumulation (seen as cytoplasmatic vacuoles) and the position of nucleus were recorded, for each measured hepatocyte, on the same microphotograph, for the classification of each liver sample into the mild, moderate, or severe lipid accumulation classes, and subsequently, for the comparison of measurements as a function of dietary treatment.

#### 2.3.2. Histopathological Evaluation

For each specimen (n = 6/dietary treatment), the presence/absence of 18 histopathological alterations (Table 2) was blindly evaluated in sea bream and sea bass livers, according to the protocol proposed by Bernet et al. [58], which has been modified for this study. Briefly, each histological alteration was checked in 9 randomly selected fields and classified into five reaction patterns: (i) circulatory disturbance, (ii) regressive changes (iii) progressive changes, (iv) inflammation, and (v) tumors. For each alteration, an importance factor (w), with a score ranging from 1 to 3 (1, minimal; 2, moderate; and 3, marked pathological importance), was assigned. An importance factor w = 1 was attributed both for mild and moderate lipid accumulation, characterized by increasing lipid vesicle deposition in the cytoplasm, and the nucleus still centrally or para-centrally located. The score w = 2 was assigned in the presence of severe lipid accumulation, with vacuoles completely filling the cytoplasm of the hepatocyte and consequent nucleus displacement at the periphery. 

Due to the uneven distribution within the hepatic tissue, the frequency percentage of histological alterations within each of the 9 fields was also considered by assigning a score ranging from 0 to 6, as follows: 0 = absent; 2 = low ≤ 10%; 4 = medium 10–50%; 6 = high ≥ 50%. Intermediate values were not considered [61]. Peri-pancreatic fat infiltration was evaluated in sea bream using the above 0–6 grading scale on three whole sections per liver at 50× magnification. This parameter was not included among the 18 histopathological alterations used for the calculation of the liver index. 

#### 2.3.3. Liver Index Calculation

A liver index formula was developed, based on the scoring system reported by Bernet et al. [58], and modified for this study. The formula considers the percentage ratio between the sum of the weighted scores of observed histological alterations and the sum of the maximum achievable weighted scores, assigning a score of 6 to all examined alterations. 

The liver index (LI) was calculated for each fish as follows:

$${LI}_{i}=\frac{\sum_{j:1}^{k}{s_{j}w}_{j}}{a\sum_{j:1}^{k}max(s_{j})w_{j}}\times 100\;\mathrm{for}\;i:1,2,\ldots ,n$$

where:

i—ith fish;
s_j_—the score assigned to each histological alteration (j) from 0 to 6, considering the frequency percentage within each field, as described above;
w_j_—the importance factor, expressing the severity of the histological alteration (j);
a—the number of observed fields;
s_j_—the maximum score attainable for each histological alteration.

This index indicates the extent and severity of liver histological alterations, enabling the conversion of qualitative observations into quantitative values.

### 2.4. Blood Biochemistry Analyses

A set of metabolic parameters, including glucose (GLU), triglycerides (TAGs), cholesterol (CHO), total protein (TP), albumin (ALB), aspartate aminotransferase (AST), and alanine aminotransferase (ALT) were analyzed on serum samples using an automated biochemistry analyzer (BPC BIOSED, Rome, Italy) and commercial kits (Giesse Diagnostics, Rome, Italy), according to Cardinaletti et al. [79].

### 2.5. Statistical Analysis

Datasets on fish size, body condition indices, hepatocyte measurements, liver index values, and blood chemistry parameters were analyzed using the Kruskal–Wallis H-test and followed by post hoc multiple comparisons with Bonferroni adjustment to evaluate the effects of the dietary treatments (*p* < 0.05). The Chi-square test was used to evaluate differences in the frequency percentage of the affected area by the histological alterations. 

In this study, focused principal component analysis (FPCA) was applied to evaluate the relationships between biometric and biochemical parameters and liver index values, with the latter considered the variable of interest, as well as to examine the existing correlations among the variables themselves. The graphical visualization of FPCA analysis shows correlations as concentric circles, with the variable of interest positioned at the center, directing the analysis. Stronger correlations are represented by smaller circles. A red circle is used to indicate statistical significance at *p* < 0.05. Negative and positive correlations are distinguished in the graph using yellow and green dots, respectively. The interpretation of existing correlations among explanatory variables follows the same criteria as classical PCA analysis. The data analysis was carried out using the R software statistical package 3.6.2 version [80].

### 2.6. Ethical Approval

The present study was approved by the Ethics Committee of the University of Udine and authorized by the Italian Ministry of Health (n.290/2019-PR), in accordance with European legal frameworks on the protection of animals used for scientific purposes (Directive 2010/63/EU).

## 3. Results

### 3.1. Biometric Measurements and Fish Condition

All of the diets were highly palatable and were well accepted. No mortality occurred throughout the trial. The growth and body condition indices of sea bream and sea bass are shown in Table 3. In sea bream, the final BW of sample specimens was significantly higher in fish fed the H40, P40, and H10P30 diets, compared to the controls (CV). The HSI values were similar in sea bream fed the CV and those fed the H40 and P40 diets, while the HSI values were significantly higher in fish fed the H10P30 diet, compared to the P40 dietary group. No significant differences were observed in final BW due to dietary treatment in sea bass. K values were significantly higher in fish fed the H10P30 diet, compared to the P40 dietary group, while no significant differences were observed in their HSI values.

Post mortem analysis showed an overall good health status for both sea bream and sea bass fed test diets. Macroscopic observation revealed a clear difference in the liver appearance between sea bream and sea bass. Sea bream showed a low-to-medium degree of lipid accumulation in all dietary groups, while a high degree was rarely observed. Indeed, the liver color was not homogeneous in sea bream specimens, ranging from dark red to light brown, with a few cases showing areas of pale color. The consistency of the organ was maintained in most of the samples that were examined. Conversely, most sea bass displayed a high degree of liver lipid accumulation in all dietary groups, while a moderate degree was observed in a few specimens and a mild degree was not found. The livers of sea bass presented diffuse areas ranging from whitish to light yellowish in color, which, in most cases, led to easy fragmentation.

**Table 3:** Total length, live weight, and body condition indices of sea bream and sea bass fed test diets over 12 and 18 weeks, respectively.

		Test Diets				Kruskal–Wallis Test
		CV	H40	P40	H10P30	
**Sea bream**	TL(cm)	21.9 ± 0.6	22.2 ± 0.8	22.3 ± 0.6	22.3 ± 0.3	n.s.
	BW final (g)	175.2 ± 17.1 ^b^	188.8 ± 24.8 ^a^	190.3 ± 15.7 ^a^	192.6 ± 9.5 ^a^	*p* < 0.05
	K (g/cm^3^)	1.67 ± 0.12	1.72 ± 0.13	1.70 ± 0.06	1.74 ± 0.07	n.s.
	HSI (%)	1.29 ± 0.27 ^ab^	1.34 ± 0.14 ^ab^	1.27 ± 0.18 ^b^	1.50 ± 0.19 ^a^	*p* < 0.01
**Sea bass**	TL (cm)	22.8 ± 1.02	23.3 ± 94	23.2 ± 1.38	23.5 ± 0.85	n.s.
	BW final (g)	160.7 ± 28.2	159.3 ± 20.4	154.5 ± 27.9	171.2 ± 23.9	n.s.
	K (g/cm^3^)	1.31 ± 0.09 ^a^	1.26 ± 0.08 ^ab^	1.22 ± 0.04 ^b^	1.32 ± 0.06 ^a^	*p* < 0.001
	HSI (%)	1.80 ± 0.29	1.60 ± 0.17	1.67 ± 0.32	1.52 ± 0.32	n.s.

Data are expressed as mean ± sd. Different superscripts indicate significant differences among dietary treatments. n.s.: not significant.

### 3.2. Liver Histology

**Figure 1:**
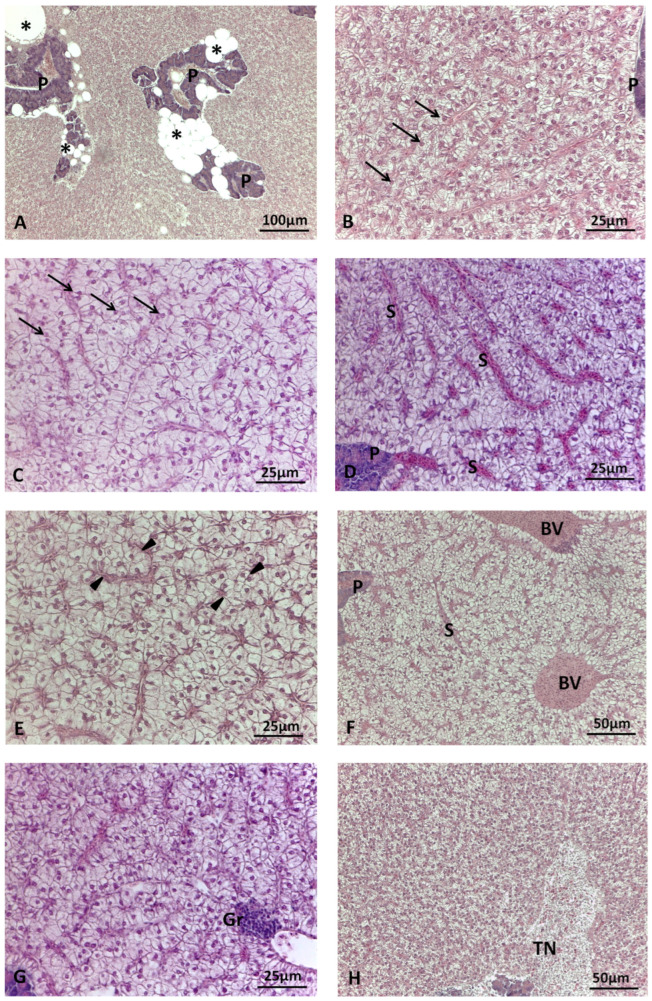
Main histological alterations in the livers of sea bream fed test diets. (**A**) CV: large areas of peri-pancreatic fat (asterisks) infiltration; (**B**) H40 diet: mild hepatocyte lipid accumulation and central round nuclei (arrows); (**C**) P40 diet: moderate hepatocyte lipid accumulation and round nuclei still centrally or para-centrally located (arrows); (**D**) P40 diet: sinusoid congestion; (**E**) H10P30 diet: hepatocyte hyperplasia and cord loss (arrowheads); (**F**) H40 diet: blood vessel congestion; (**G**) P40 diet: focus of granulocytes; (**H**) P40 diet: tissue necrosis area. BV = blood vessel; P = exocrine pancreas; Gr = granulocytes; S = sinusoid; TN = tissue necrosis. Hematoxylin and eosin staining.

In most cases, the structure of the hepatic parenchyma exhibited irregular cords, arranged in two cellular layers of hepatocytes surrounded by sinusoids. The roundish polygonal hepatocytes appeared moderately eosinophilic and displayed a clear spherical nucleus, positioned centrally or peripherally depending on the degree of lipid accumulation. The occurrence of alterations, which varied in extent and severity according to diet and species, and which were mostly associated with lipid accumulation, is detailed in Section 3.2.2. In both species, the exocrine pancreatic tissue appeared to be well organized within the hepatic structure, surrounding the branches of the hepatic portal vein. A medium- to high-frequency percentage of peripancreatic fat infiltration was observed in sea bream (Figure 1A), without any significant differences among dietary groups. 

#### 3.2.1. Histomorphometry 

In sea bream, the mean area of hepatocytes with moderate lipid accumulation was significantly higher (165.4 µm^2^) than in hepatocytes with mild lipid accumulation (97.4 µm^2^), while the nucleus area and the nucleus/hepatocyte ratio were significantly smaller (Table 4). Similarly, in sea bass, the mean cell area significantly increased based on mild (119.1 µm^2^), moderate (205.3 µm^2^), or severe (372.1 µm^2^) lipid accumulation. In this species, a significant decrease in mean nucleus area was only observed between mild and severe lipid accumulation, while a strong significant decrease was observed for the nucleus/hepatocyte ratio among all classes of lipid accumulation.

Histomorphometric differences in hepatocytes were also observed as a function of dietary treatment (Table 5). In sea bream and sea bass, the mean cell area was significantly larger in the dietary replacement groups compared to the CV group, while the mean nucleus area and nucleus/hepatocyte area ratio were significantly smaller. The largest hepatocytes were found in sea bream fed the H10P30 diet (177.9 ± 50.5 µm^2^) and in sea bass fed the P40 diet (428.1 ± 23.5 µm^2^), compared to the other dietary treatments.

**Table 4:** Histomorphometric measurements of hepatocytes as a function of lipid accumulation levels in sea bream and sea bass (n = 90 cells per fish).

		Lipid Accumulation			Kruskal–Wallis Test
		Mild	Moderate	Severe	
**Sea bream**	Hepatocyte area (µm^2^)	97.4 ± 13.4 ^a^	165.4 ± 37.9 ^b^	-	*p* < 0.001
	Nucleus area (µm^2^)	16.5 ± 1.8 ^a^	13.0 ± 1.2 ^b^	-	*p* < 0.001
	N/H ratio	0.17 ± 0.03 ^a^	0.08 ± 0.02 ^b^	-	*p* < 0.001
**Sea bass**	Hepatocyte area (µm^2^)	119.1 ± 17.1 ^a^	205.3 ± 40.1 ^b^	372.1 ± 175.1 ^c^	*p* < 0.001
	Nucleus area (µm^2^)	15.5 ± 0.36 ^a^	14.6 ± 1.4 ^a^	12.32 ± 1.3 ^b^	*p* < 0.001
	N/H ratio	0.13 ± 0.02 ^a^	0.07 ± 0.02 ^b^	0.04 ± 0.01 ^c^	*p* < 0.001

Data are expressed as mean ± sd. Different superscripts indicate significant differences among groups.

#### 3.2.2. Histopathology 

**Figure 2:**
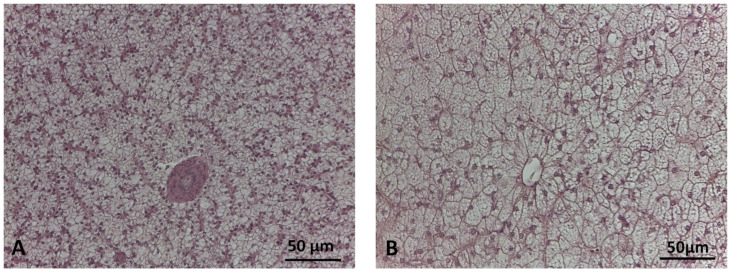

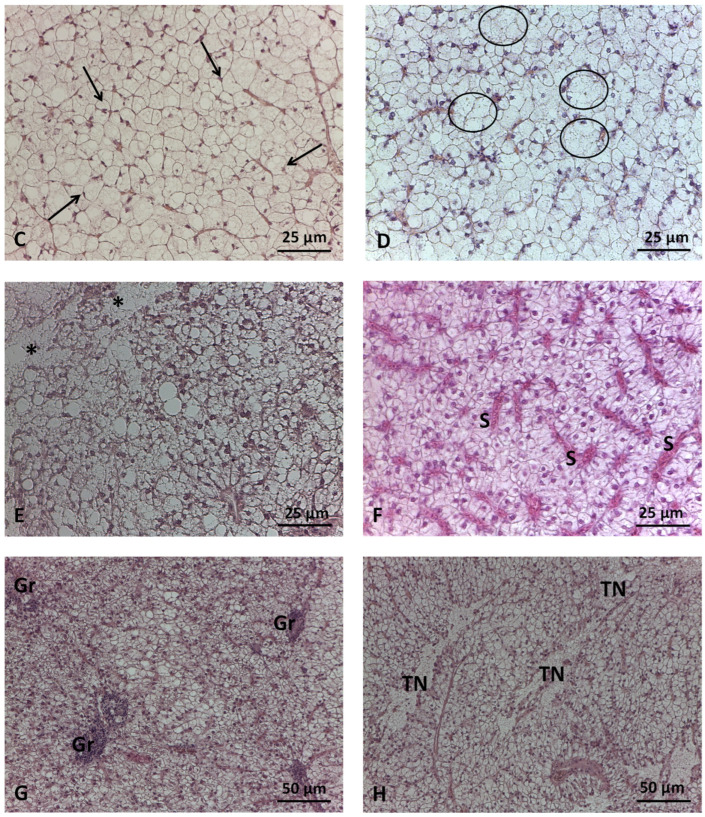
Main histological alterations in the livers of sea bass fed test diets. (**A**) CV: moderate hepatocyte lipid accumulation; (**B**) P40 diet: severe hepatocyte lipid accumulation; (**C**) P40 diet: peripheral nuclei in hepatocytes with severe lipid accumulation (arrows); (**D**) H10P30: hepatocyte hypertrophy (circles); (**E**) P40 diet: vacuolar degeneration (asterisks) areas and cord loss; (**F**) H10P30 diet: sinusoid congestion; (**G**) P40 diet: foci of granulocytes; (**H**) P40 diet: tissue necrosis areas. S = sinusoids; Gr = granulocytes; TN = tissue necrosis. Hematoxylin and eosin staining.

**Figure 3:**
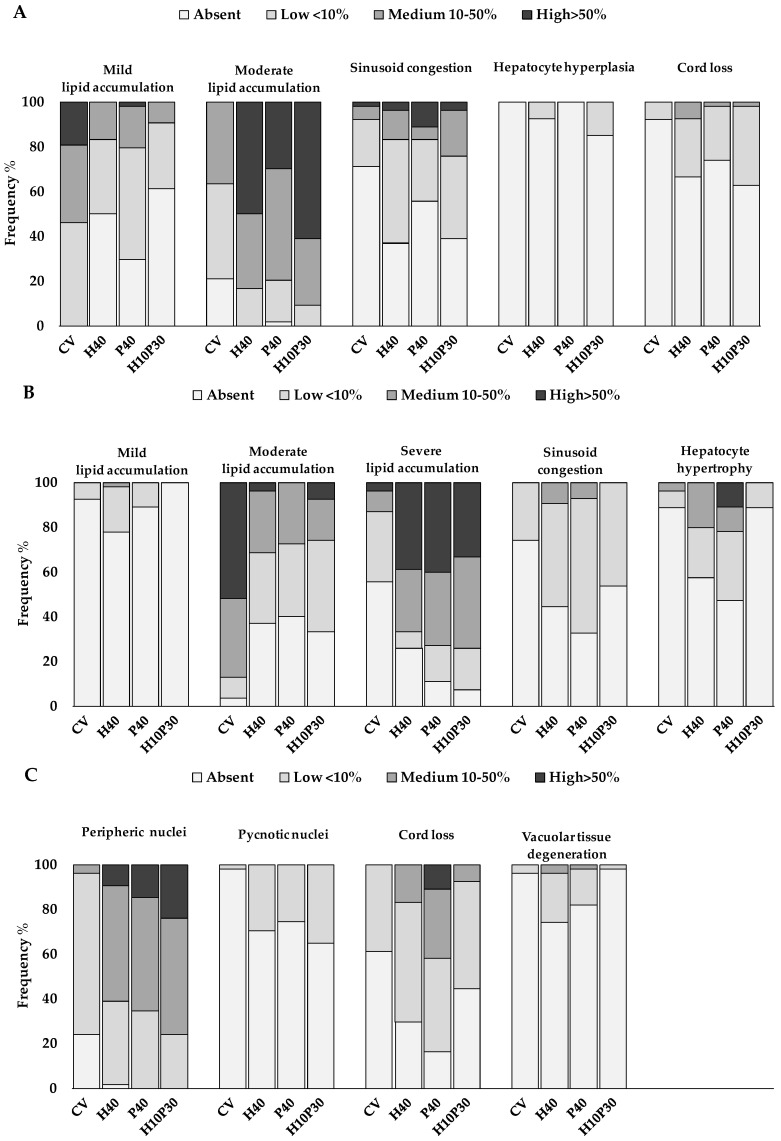
Frequency percentages of significant histological alterations in the livers of sea bream (**A**) and sea bass (**B**,**C**) fed test diets. Chi-square test, *p* < 0.05.

The main histological alterations observed in sea bream and sea bass livers are illustrated in Figure 1 and Figure 2, while the frequency percentages of significant histological alterations are presented in Figure 3. In sea bream, significant differences in response to dietary treatments were observed for hepatocyte lipid accumulation (*p* < 0.001), sinusoid congestion (*p* < 0.005), cell hyperplasia (*p* < 0.05), and cord loss (*p* < 0.01). Mild and moderate lipid accumulation were observed in the livers of sea bream (Figure 1B,C). Moderate lipid accumulation was prevalent in fish fed replacement diets compared to the CV. Considering the frequency percentages greater than 10% (i.e., the sum of scores 4 and 6) for this alteration, the frequencies observed were 83.3% in the H40 group, 79.6% in the P40 group, and 90.7% in the H10P30 group, compared to the CV group (37%) (Figure 3A). Sinusoid congestion was prevalent in sea bream fed the H40 (63%), H10P30 (61.1%), and P40 (44.5%) diets (Figure 1D), compared to the CV (29%). A low frequency of cell hyperplasia (14.8%) (*p* < 0.05) and moderate cord loss (35%) were also observed, particularly in sea bream fed the H10P30 diet (Figure 1E). No significant differences were observed in blood vessel congestion (12.0%, 9.3%, 9.3%, and 16.7% in the CV, H40, P40, and H10P30 groups) (Figure 1F), nor in granulocyte occurrence (13%, 11.3%, 14.8%, and 5.6% in the CV, H40, P40, and H10P30 groups) (Figure 1G). Tissue necrosis was observed in all dietary groups in a non-significant manner (Figure 1H).

Sea bass showed a worse overall histopathological picture compared to sea bream, in terms of the number and severity of the histological alterations observed (Figure 3B,C). The CV group was characterized by moderate lipid accumulation (87%) (*p* < 0.001) (Figure 2A), while fish fed test diets were mainly characterized by severe lipid accumulation (66.7%, 72.7%, and 74.0% in the H40, P40, and H10P30 groups, respectively) (*p* < 0.001) (Figure 2B). Additional alterations related to severe lipid accumulation were observed in sea bass, including nucleus peripheral displacement (Figure 2C), hepatocyte hypertrophy (Figure 2D), cord loss (Figure 2E) (*p* < 0.001), and tissue vacuolar degeneration (*p* < 0.01), accounting for 100%, 52.7%, 80.6%, and 18.2%, respectively, in the P40 diet compared to the others. This group also presented a higher frequency percentage of sinusoid congestion (52.7%) (*p* < 0.001) (Figure 2F) and a limited frequency percentage (5.4%) of granulocytes, without significant differences among dietary groups (Figure 2G). Only a few pyknotic nuclei were observed in sea bass groups fed the H40, P40, and H10P30 diets, but their frequency was higher with respect to the CV (*p* < 0.001). Tissue necrosis occurred with low frequency and did not significantly differ among groups (Figure 2H). Bile ducts were patent in all groups, and no hemorrhagic pattern was ever observed either in sea bream or sea bass livers.

### 3.3. Liver Index

#### 3.3.1. Formula and Scoring Scheme 

The LI formula is based on the percentage ratio between the histological alterations observed and a theoretical reference condition. This reference condition is derived by assigning the maximum score of 6 to all alterations observed in the nine fields, representing the most severe histopathological condition observable. For this reason, the proposed formula for the liver index returns lower values compared to those calculated using the Bernet index. The two indices were compared through a regression analysis. The mathematical model that links the two indices has zero intercept, the same angular coefficient, 0.67, for both sea bream and sea bass, and the coefficient R^2^ ~ 1. Based on the slope factor, the LI values were recalibrated according to the scoring scheme of Zimmerli et al. [60], and five corresponding classes were identified (Table 6).

#### 3.3.2. Liver Health Calculation

**Figure 4:**
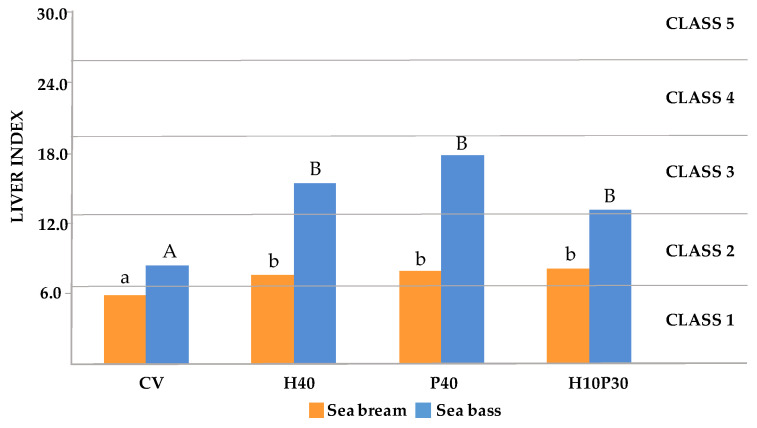
Liver index of sea bream and sea bass fed test diets. Data are expressed as mean ± sd. Different superscripts indicate significant differences among groups within the same species (*p* < 0.05).

The LI values are shown in Figure 4. Dietary treatment significantly affected the LI values of both sea bream (*p* < 0.001) and sea bass (*p* < 0.01). In both species, the LI values were significantly higher in fish fed the H40, P40, and H10P30 diets, compared to the CV (*p* < 0.05). The LI values in sea bream varied, from 5.8 in the CV group (Class 1), to 7.6, 7.9, and 9.2, respectively, in the H40, P40, and H10P30 groups (Class 2). The LI values were higher in sea bass, varying from 8.4 in the CV group (Class 2), to 15.5, 17.9, and 13.2, respectively, in the H40, P40, and H10P30 groups (Class 3), indicating a more pronounced dietary effect on liver health in sea bass compared to in sea bream. In particular, sea bass fed the P40 diet showed the highest LI value, although it was not significantly different from those of the H40 and H10P30 dietary groups. 

### 3.4. Blood Biochemistry

**Figure 5:**
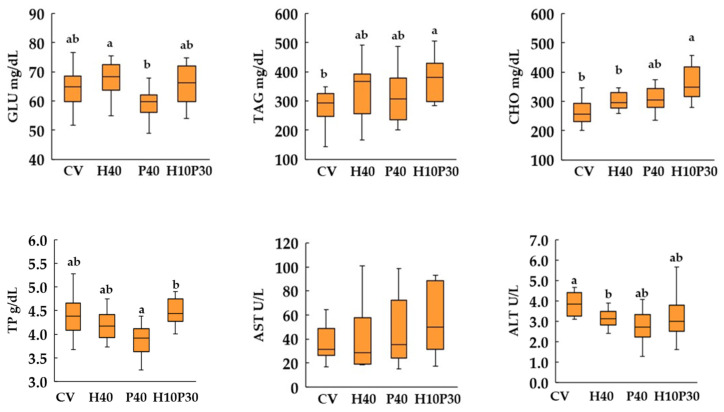
Box plot of serum biochemical parameter levels in sea bream fed test diets. Different superscripts indicate significant differences among dietary treatments. The data expressed are the median, lower and upper quartiles, and minimum and maximum values. GLU = glucose; TAG = triglycerides; CHO = cholesterol; TP = total protein; AST = aspartate aminotransferase; ALT = alanine aminotransferase.

**Figure 6:**
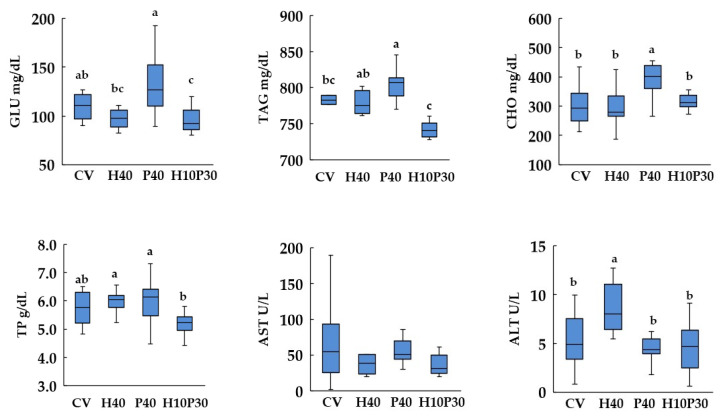
Box plot of serum biochemical parameter levels in sea bass fed test diets. Different superscripts indicate significant differences among dietary treatments. The data expressed are the median, lower and upper quartiles, and minimum and maximum values. GLU = glucose; TAG = triglycerides; CHO = cholesterol; TP = total protein; AST = aspartate aminotransferase; ALT = alanine aminotransferase.

Dietary treatment significantly affected serum biochemical parameters, except AST enzyme activity, in both species (*p* < 0.05) (Figure 5 and Figure 6). In sea bream, the serum GLU and TP levels in the P40 dietary group were similar to those of the CV group and significantly lower (*p* < 0.05) compared to the levels measured in the H10P30 group. These parameters remained similar between sea bass fed the P40 diet and the control group, but were significantly higher compared to the H10P30 dietary group. The serum levels of CHO and TAG showed a progressive increase in sea bream fed with the H40, P40, and H10P30 diets, compared to the group fed the CV, with results significantly higher in only the H10P30 dietary group, compared to the CV group. In sea bass, these parameters were significantly higher only in fish fed the P40 diet, compared to the CV (*p* < 0.05). The lowest levels of TAG were measured in sea bass fed the H10P30 diet. Furthermore, sea bass exhibited notably higher TAG levels (700–800 mg/dL) compared to sea bream (300–400 mg/dL), regardless of dietary treatment. The ALT enzyme activity was significantly lower in sea bream and higher in sea bass fed the H40 diet compared to the CV.

**Figure 7:**
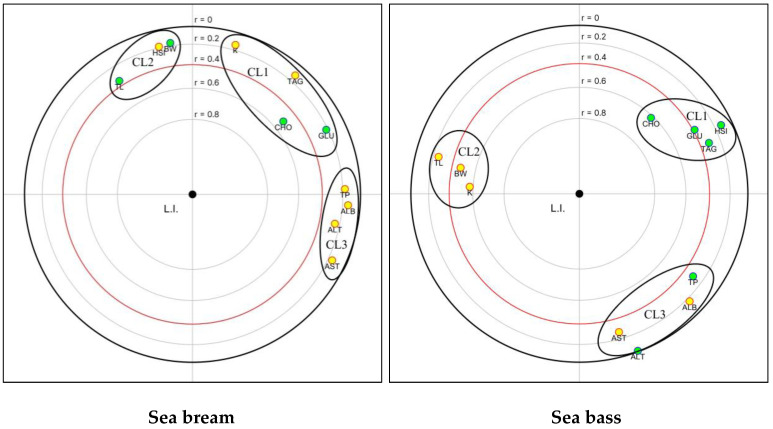
Plots of FPCA analysis on the liver index, biometric, and biochemical parameters in sea bream and sea bass. GLU = glucose; TAG = triglycerides; CHO = cholesterol; TP = total protein; AST = aspartate aminotransferase; ALT = alanine aminotransferase. Parameters inside the red circle are correlated with the liver index (LI). Green dot = positive correlation; yellow dot = negative correlation.

An FPCA analysis was applied to examine the relationships between the biochemical parameters and LI values in sea bream and sea bass (Figure 7). Cholesterol was significantly correlated with the LI (visualized inside the red circle) in both species. Moreover, significant inverse correlations were evident between the LI values and both the BW and K values in sea bass. Three clusters (CLs) of variables that showed positive correlations were identified in both species. The first cluster comprised CHO, GLU, and TAG in sea bream, along with the HSI in sea bass. In sea bream, the HSI showed a strong association with the BW and TL (second cluster). Notably, metabolic parameters grouped in cluster 1 displayed an inverse correlation with cluster 2 variables (BW, TL, and K) in sea bass, resulting in an opposite positioning. The third cluster consisted of TP, ALB, AST, and ALT, which exhibited closer relationships in sea bream than in sea bass.

## 4. Discussion 

Ensuring fish health and welfare has become a priority in modern research on fish nutrition and feeding in aquaculture, in order to improve the sustainability of feed industry, as well as the profitability and ethics of aquacultural production [81]. Assessing fish health and welfare is particularly significant in feeding trials aimed to test alternative diets, as sub-optimal feed formulations can impact fish physiology and adversely affect their growth performance, metabolites, and immune systems [7,82,83,84]. Blood biochemistry is an effective method for assessing the health and welfare of farmed fish. This method is valuable for both farming management and research applied to fish nutrition and feeding, as it enables the early identification of changes in physiological parameters related to alterations in organ functions before pathological conditions occur [41,85,86]. The liver is a key metabolic organ and an important indicator of nutritional pathology [47]. Histological investigations represent an initial step in detecting potential changes associated with inflammation, degeneration, and cell death. These investigations are crucial for assessing the effects of novel diet formulations in feeding trials on liver metabolism and health [48,49,79]. 

This study highlighted different responses to the test diets between sea bream and sea bass, in terms of biometric and blood chemistry parameters, as well as in liver health status. In sea bream, the dietary inclusion of alternative ingredients resulted in a significant increase in final body weight and somatic indices, indicating beneficial effects on overall fish health. These findings are supported in the FPCA analysis, which revealed positive correlations between the HSI and both BW and TL. Conversely, these effects were not observed in sea bass, in whom final BW remained unchanged among dietary groups, and the values of K decreased, especially in the P40 dietary group. The values of K and the HSI indices observed in this study for sea bream and sea bass are consistent with those reported in the literature for healthy fish [34,35]. Serum biochemical parameters remained within the physiological range across all dietary groups, suggesting good nutritional status in both fish species [87,88]. Major changes were observed in serum CHO and TAG levels, which progressively increased in sea bream fed the H10, P40, and H10P30 diets compared to fish fed the CV. In sea bass, these changes were only evident in fish fed the P40 diet, despite very high levels of TAG found in all dietary groups. The observed increase in serum lipid levels may be in response to changes in the relative proportion of plant protein-rich feed mixture in the diets, including the test ingredients. This could reflect a reduced lipotropic effect, due to the concurrent lower inclusion levels of soy products [86,89,90,91]. The differences in CHO and TAG levels observed among the H40, P40, and H10P30 dietary groups can be partially explained by higher intakes of digestible carbohydrates, due to the different starch contents in the replacement diets compared to that in the control diet. Additionally, the effects of chitin and taurine, present in insect meal, may have contributed to minor differences among fish fed the replacement diets [27,31,37,38,88], as well as differences in the amino acid compositions of proteins from insect and poultry by-product meals, could also have affected de novo lipid synthesis, contributing to increased serum lipid levels [91,92,93]. Indeed, the carbohydrate levels in the diet and lipid sources, as well as the proportion of saturated and unsaturated fatty acids, regulate glucose and lipid metabolism in sea bream and sea bass [94,95,96]. The positive correlation obtained from FPCA analysis, among GLU, CHO, and TAG levels in both fish species, indicates a relationship between carbohydrate and lipid metabolism, supporting this hypothesis. These parameters were further correlated with K values in sea bream and with HSI in sea bass, suggesting different metabolic statuses in the two fish species, favoring growth in the former and accumulation of liver lipids in the latter. The histological results, which are detailed below, also provide support for this hypothesis. 

According to the FPCA analysis, serum TP was only correlated with serum transaminases, which showed very low values across diets. The AST enzymatic activity was unaffected by the dietary treatment, while minor changes were observed in ALT activity in sea bass fed the H40 diet, compared to the other groups. High liver ALT activity was reported in sea bream fed the diet containing 30% *H. illucens* meal and was interpreted as increased amino acid catabolism for energy production [34].

Histological observations highlighted the presence of several alterations, primarily associated with lipid accumulation, with lower numbers and frequency percentages in sea bream than in sea bass, indicating an overall better liver condition in sea bream. These findings confirm the macroscopic observations, which indicated low-to-medium levels of hepatic lipid accumulation for sea bream and medium-to-high levels for sea bass. These data also appear consistent with the histomorphometric analysis. Indeed, the mean values of hepatocyte and nucleus areas, as well as the N/H ratio, correspond to mild-to-moderate hepatocyte lipid accumulation for sea bream and from mild to severe hepatocyte lipid accumulation for sea bass. Furthermore, the histomorphometric evaluation highlighted significant differences among the tested diets, consistently correlated with the severity and frequency of the observed hepatic lipid accumulation in both fish species. In this regard, the mean hepatocyte area was significantly larger in sea bream fed the H10P30 diet, a result which is in agreement with the higher frequency of moderate lipid accumulation compared to fish fed the H40 and P40 diets. In sea bass, the mean hepatocyte area was significantly larger in fish fed the H40, P40, and H10P30 diets, compared to those fed the CV. The largest mean area was measured in the hepatocytes of P40-fed fish, which corresponds to the highest frequency percentage of severe lipid accumulation and vacuolar tissue degeneration. These results confirm the utility of the histomorphometric analysis in assessing and comparing the effects of different feeds on fish livers, as previously reported by Raskovic et al. [97]. However, our results are not directly comparable to those reported by other authors, as they were calculated based on different measurements of hepatocytes and nuclei, such as the diameter ratio in sea bream [72] and sea bass [70], or the maximum and minimum length of hepatocytes, considering the nucleus as an arbitrary reference point in sea bream fed microalgae and poultry oils [71].

Among the 18 histopathological parameters selected for this study, those most frequently observed to be directly related to diet were those associated with the degree of lipid accumulation, which showed different frequencies among dietary groups and between the two fish species. Compared to the control group, the hepatocytes of sea bream fed the replacement diets, in addition to higher lipid accumulation, also displayed low-to-medium occurrence of sinusoidal congestion, hyperplasia, and cord loss. Similar results were reported in the same fish species fed a diet in which 50% fish meal was replaced with poultry by-product meal [29]. Hepatic lipid accumulation is classified as a regressive histological alteration. Its reversibility was observed in sea bream fed vegetable oils, when fish were re-fed with a balanced diet, denoting the non-pathological character of this liver alteration [54]. The hepatocyte hyperplasia observed in sea bream fed the H40 and H10P30 diets could, in part, be interpreted as an adaptive response to increased carbohydrate metabolism. The higher, although not statistically significant, serum glucose levels observed in these dietary groups, compared to the P40 group, provide support for this interpretation. Hepatocyte hyperplasia has also been reported in relation to a faster absorption rate of carbohydrates from pea seed meal in sea bass [51]. These histological findings are consistent with the higher HSI values and serum lipid levels, and they were particularly observed in sea bream fed the H10P30 diet, who showed a higher frequency of moderate lipid accumulation and hepatocyte hyperplasia, compared to the CV group. Moderate-to-severe peri-pancreatic fat infiltration was observed in sea bream, with no significant differences among dietary groups. This seems consistent with the histological picture reported in this fish species after long-term feeding with vegetable oils [54]. On the contrary, Baeza-Ariño et al. [56] observed a significant reduction in fat infiltrations in sea bream fed a mixture of vegetable protein concentrates vs. a commercial diet.

In sea bass, the hepatocyte lipid accumulation was found to be mostly moderate in fish fed the control diet, but turned to severe in those fed the replacement diets. Other significant associated alterations were observed in the latter dietary groups, including the displacement of the peripheral nucleus, hepatocyte hypertrophy, cord loss, tissue vacuolar degeneration, pycnotic nuclei, and sinusoid congestion, which are indicative of an incipient steatotic condition, probably due to altered liver lipid metabolism. A worsening of the liver health condition was observed in sea bass fed the P40 diet, which exhibited the most pronounced histological alterations. These alterations appear to be slightly attenuated in fish fed the H10P30 diet, consistently observed with lower serum TAG levels, compared to the P40 dietary group. Liver steatosis generally occurs when dietary lipids, or energy, exceed the capacity of hepatic cells to oxidize fatty acids, resulting in increased TAG synthesis and deposition into hepatocytes [98,99]. The balance between lipogenesis and lipolysis is maintained through various transcription factors and genes, which, in turn, are regulated by dietary fatty acids [71,83,100]. Steatotic liver is generally considered a paraphysiological condition, commonly detectable in farmed sea bass and sea bream in relation to dietary regimes [65,101,102,103].

The results of LI analysis also highlight a major influence of the dietary treatment on liver health status in both fish species, but with a different amplitude. In sea bream, slightly but significantly higher LI values were observed in fish fed the H40, P40, and H10P30 diets, indicating slight modifications to normal liver architecture and morphology (Class 2), compared to those fed the control diet (who fell into Class 1, normal liver histology), which is consistent with histomorphometric results. Higher LI values, falling into Class 3 and indicating the occurrence of moderate liver alterations, were found in sea bass fed replacement diets, compared to fish fed the CV, who displayed an LI value in Class 2. In any case, these results indicate that the liver parenchyma architecture and its functional condition were maintained. These findings are consistent with the biochemical and histological observations, particularly the elevated serum TAG levels and severe lipid accumulation observed in all dietary groups. The results of the FPCA highlighted a significant correlation between the LI values and CHO levels (the green dot inside the red circle in Figure 7) in both sea bream and sea bass. This suggests that the changes in dietary CHO are the primary causes of the metabolic effects observed when replacing plant proteins with insect and poultry by-product meals in the diet. However, the significant inverse correlations between LI values and both final BW and K values in sea bass (the yellow dots inside the red circle in Figure 7) suggest potential influences of liver condition on fish body weight and biometric parameters. 

The developed histological protocol, new LI formula, and scoring scheme, which enabled the transformation of qualitative observations into a semi-quantitative index, provided objective information on the liver health of sea bream and sea bass. Particular attention has been paid to evaluating the severity and frequency of hepatocyte lipid accumulation, and the associated alterations, in response to dietary treatments. Differently from the Bernet index [58], the new LI formula and the developed scoring scheme express the observed liver health condition in relation to a theoretical reference condition, making the assessment more objective, compared to the criterion used by Zimmerli et al. for defining the severity classes of the liver index [60]. Moreover, based on the results of regression analysis, the new LI formula returns values equivalent to those of the Bernet index, and the recalibrated classes are equivalent to those developed by Zimmerli et al. [60], allowing for the comparison of our results with the literature data. 

The biochemical and histological results indicated a favorable physiological status and good liver conditions in sea bream, with slight increases in serum CHO and TAG levels, along with moderate hepatocyte lipid accumulation, especially in response to the H10P30 diet. In sea bass, elevated serum TAG levels and severe liver lipid accumulation suggest high energy contents in the replacement diets under the experimental conditions [82,104,105]. This could partly reflect the high dietary lipid content and/or increased saturated fatty acid intake with diets including the test ingredients, particularly the *H. illucens* meal, whose lipid moiety is characterized by high levels of saturates and a high saturated/polyunsaturated ratio [43]. However, this condition did not influence body weight or nutritional status. High levels of circulating lipids, as well as fatty liver, often occur when farmed fish are fed moderate- to high-energy commercial feeds, similar to those formulated in the present study [102,106].

## 5. Conclusions

Overall, the results of this study highlighted species-specific differences in the physiological responses to alternative dietary protein-rich ingredients. Sea bream adapted to the replacement diets, displaying efficient utilization of dietary nutrients, which resulted in improved body weight gain and good liver health. Sea bass, despite the HSI values observed in this study being consistent with those reported in the literature for healthy fish, showed altered liver lipid metabolism and additional lipid accumulation when fed diets rich in *H. illucens* and poultry by-product meals. This led to incipient hepatic steatosis. Therefore, potential risks to liver health cannot be excluded when fish are fed for longer durations with substantial levels of processed animal proteins in their diets.

In this study, blood biochemistry and liver histomorphology proved to be highly informative in assessing the physiological statuses of sea bream and sea bass in response to the tested diets. This stresses the importance of evaluating liver health for an overall assessment of fish welfare in relation to feeding treatments. Moreover, consistent with biochemical and histological findings, the liver index formula proposed herein proved to be a reliable tool for assessing the liver health of both sea bream and sea bass. 

## Figures and Tables

**Table 1:** Ingredient (g 100 g^−1^) and proximate (% as fed) compositions of the test diets.

Ingredient Composition (g 100 g^−1^)	CV	H40	P40	H10P30
Vegetable protein mix ^1^	69.0	36.6	35.4	35.4
*Hermetia illucens* meal ^2^	-	32.4	-	8.1
Poultry by-product meal ^3^	-	-	27.5	20.6
Feeding stimulants ^4^	5.5	5.5	5.5	5.5
Wheat meal *	0.4	4.5	5.6	5.5
Whole pea *	3.0	6.0	9.0	8.8
Fish oil ^5^	6.2	6.2	6.2	6.2
Vegetable oil mix ^6^	11.4	5.4	8.2	7.4
Vit. & Min. Premix ^7^	0.4	0.4	0.4	0.4
Sodium phosphate	1.6	1.0	0.3	0.2
L-Lysine ^8^	0.5	0.2	0.1	0.1
DL-Methionine ^9^	0.5	0.3	0.3	0.3
Celite	1.5	1.5	1.5	1.5
**Proximate composition (% as fed):**				
Moisture	6.7	6.0	7.1	8.7
Protein (Nx6.25)	45.0	45.2	45.1	45.1
Total lipid	20.4	20.4	20.3	20.4
Ash	5.8	6.6	7.8	7.7
Chitin ^#^	0.02	1.54	0.02	0.40
Energy (MJ/kg)	21.5	21.0	21.7	21.5

^1^ Vegetable protein source mixture (% composition): dehulled, toasted soybean meal, 39; soy protein concentrate-Soycomil, 20; maize gluten, 18; wheat gluten, 15; rapeseed meal, 8. ^2^ ProteinX™, Protix, Dongen, The Netherlands. ^3^ Poultry by-product meal from Azienda Agricola Tre Valli; Verona, Italy. ^4^ Feeding stimulants, g/100 diet: CPSP90, Sopropêche; Wimille, France, 3.5; Squid meal, 2.0. ^5^ Fish oil: Sopropêche; Wimille, France. ^6^ Vegetable oil mixture (% composition): rapeseed oil, 56; linseed oil, 26; palm oil, 18. ^7^ Supplied per kg of vitamin supplement: Vit. A, 4,000,000 IU; Vit D3, 850,000 IU; Vit. K3, 5000 mg; Vit. B1, 4000 mg; Vit. B2, 10,000 mg; Vit B3, 15,000 mg; Vit. B5, 35,000 mg; Vit B6, 5000 mg; Vit. B9, 3000 mg; Vit. B12, 50 mg; Biotin, 350 mg; Choline, 600 mg; Inositol, 150,000 mg. Supplied per kg of mineral supplement: Ca, 77,000 mg; Cu, 2500 mg; Fe, 30,000 mg; I, 750 mg; Se, 10,000 mg; Zn, 25 mg. ^8^ L-lysine sulphate, 99%; Ajinomoto EUROLYSINE S.A.S; Paris, France. ^9^ DL-Methionine: 99%; EVONIK Nutrition & Care GmbH; Essen, Germany. * Wherever not specified, the ingredients comprising the diets were obtained from local providers by Sparos Lda. ^#^ Estimated based on chitin content of the ingredients used (squid meal, 0.9% and *Hermetia illucens* meal, 4.69%).

**Table 2:** Histological alterations (j) and importance factors (w) considered for histopathological liver evaluation in sea bream and sea bass fed test diets.

Reaction Pattern	Histological Alteration (j)	Importance Factor (w)
Circulatory disturbance	Sinusoid congestion	w = 1
	Blood vessel congestion	w = 1
	Hemorrhages	w = 2
Regressive changes	Mild-to-moderate lipid accumulation	w = 1
	Severe lipid accumulation	w = 2
	Peripheric nuclei	w = 1
	Pycnotic nuclei	w = 2
	Cord loss	w = 2
	Vacuolar tissue degeneration	w = 2
	Tissue necrosis	w = 3
Progressive changes	Hepatocyte hyperplasia	w = 2
	Hepatocytes hypertrophy	w = 2
	Bile duct hypertrophy	w = 1
Inflammation	Granulocyte infiltration	w = 2
	MMc occurrence	w = 1
Tumor	Benign tumor	w = 2
	Malignant tumor	w = 3

**Table 5:** Histomorphometric measurements of hepatocytes based on dietary treatment in sea bream and sea bass (n = 90 cells per fish).

		Test Diets				Kruskal–Wallis Test
		CV	H40	P40	H10P30	
**Sea bream**	Hepatocyte area (µm^2^)	109.9 ± 26.8 ^a^	146.5 ± 33.1 ^b^	135.7 ± 38.8 ^b^	177.9 ± 50.5 ^c^	*p* < 0.001
	Nucleus area (µm^2^)	16.2 ± 2.4 ^a^	13.8 ± 1.2 ^b^	14.1 ± 1.9 ^b^	12.6 ± 1.2 ^c^	*p* < 0.001
	N/H ratio	0.16 ± 0.05 ^a^	0.10 ± 0.03 ^b^	0.12 ± 0.05 ^b^	0.08 ± 0.03 ^c^	*p* < 0.001
**Sea bass**	Hepatocyte area (µm^2^)	199.2 ± 44.9 ^a^	277.4 ± 89.6 ^b^	428.1 ± 23.5 ^c^	247.6 ± 56.5 ^b^	*p* < 0.001
	Nucleus area (µm^2^)	15.5 ± 0.9 ^a^	13.32 ± 1.6 ^b^	11.87 ± 1.0 ^c^	13.26 ± 1.5 ^b^	*p* < 0.001
	N/H ratio	0.08 ± 0.02 ^a^	0.06 ± 0.03 ^b^	0.03 ± 0.01 ^c^	0.06 ± 0.02 ^b^	*p* < 0.001

Data are expressed as mean ± sd. Different superscripts indicate significant differences among groups.

**Table 6:** Scoring scheme of the liver index developed for sea bream and sea bass.

	Scoring Scheme	Description
Class 1	≤6.5	Normal liver structure
Class 2	6.6–13.0	Normal liver structure with slight histological alterations
Class 3	13.1–19.5	Normal liver structure with moderate histological alterations
Class 4	19.6–25.9	Pronounced alterations of liver structure
Class 5	>26	Severe alterations of liver structure

## Data Availability

Data are contained within the article.
